# Diseases of the Maxillary Sinus

**Published:** 1895-05

**Authors:** Harrison Allen

**Affiliations:** Philadelphia


### International Dental Journal.

VOL. XVI. MAY, 1895. No. 5.

### Original Communications.[1]

### DISEASES OF THE MAXILLARY SINUS.[2]

BY HARRISON ALLEN, M.D., PHILADELPHIA.

THERE is probably no subject in clinical medicine which has undergone greater change in the last few years than that of diseases of the maxillary sinus. Prior to the time when we obtained aid by reflected light in inspecting the nasal chambers, and to the use of the electric lamp in transillumination, the diseases of the sinus were not detected in their early stages.

The older writers said, for example, that one of the signs upon which physicians must depend for diagnosing pus in the sinus is a thinning of the anterior wall. This was said to be pushed forward and to crackle under pressure of the finger at the canine fossa. Another sign relied upon was the fulness over the sinus and general swelling from the eye downward; some, indeed, spoke of the elevation of the floor of the orbit; in a word, all the tests depended upon the supposition that the chamber itself was overdistended. Thus, Salter (Holme's "System of Surgery," 1864, vol. iv. p. 27) gives the local symptoms as follows: "The expansion of the whole jaw; the fossa beneath it full and prominent; the molar teeth on the affected side appear to elongate; the concavity of the hard

[1] The editor and publishers are not responsible for the views of authors of papers published in this department, nor for any claim to novelty, or otherwise, that may be made by them. No papers will be received for this department that have appeared in any other journal published in the country.

[2] Read before the Academy of Stomatology, March 12, 1895.

palate becomes flat or even convex; the nostril on the corresponding side is more or less closed; in severe or protracted cases the floor of the orbit becomes so pushed up as to protrude the eye."

Erichson ("Science and Art of Surgery," American edition, p. 709) describes the cheek in "dropsy of the antrum" as being round and prominent and indolent, a semi-elastic tumor forming in it and protruding out of it and giving rise to the egg-shell or parchment crackling on pressure.

In Gross's "System of Surgery" the description both of the inflammation and suppuration of the maxillary sinus is denotive of graver conditions than I am aware are ordinarily met with; at least, such states as those described by Gross are occasionally seen, but that they do not express the phase of diseased action met with in rhinological practice I am convinced.

In common with all other physicians who are interested in diseases of the nose and adjacent parts I have seen many cases of maxillary disease, and I may say in a single instance only were the signs enumerated above present. The older writers evidently had in view a distinct condition.

Speaking entirely from the results of my own experience, I would say that diseases of the maxillary sinus are of five kinds, mentioned herewith, in the order of their frequency: First, a secondary pus-formation dependent upon irritation at the root of a tooth. Second, a purulent inflammation seen in rheumatic subjects and not to be separated from exhibitions of rheumatism seen elsewhere in the economy. Third, extension of empyema of the frontal sinus into the maxillary sinus. Fourth, polypoid degeneration of the mucous lining of the sinus. Fifth, presence of sero-mucus formed under catarrhal excitement.

*First.*—Pus in the maxillary sinus when arising from irritation at the root of a tooth is the condition which associates this disease with dental practice in a conspicuous manner. It is important to remember that a very slight amount of pus will sooner or later tend to find its way into the nose, so that a purulent discharge in the nostril should immediately lead the observer to examine the teeth, and if any of the molar or bicuspid series be found defective, they should receive prompt attention. Extraction of the offending teeth will cure the purulent discharge in a very short time provided that no degenerate processes are set up in the mucous membrane or bone. It will sometimes happen that after teeth have been extracted and the pus collection entirely removed, relapses will occur. In such cases we have one of three conditions to study,—irritation from imperfect absorption of the bone about the bottom of the old socket, imperfect healing of the wound created by extraction, or the presence of inspissated masses (the products of inflammation) in the chamber itself.

*Second.*—The results of dental irritation may be present in persons of a rheumatic diathesis. But inflammation of any kind, whether excited by disease about the root of a tooth or not, may extend rapidly into the sinus. Such cases are more apt to occur in middle life than at any other period, and I have reason to believe they are more common in those with history of alcoholic excess than in the temperate.

Rheumatic inflammation may occur on both sides of the face.

*Third.*—Disease of the frontal sinus if neglected is apt to extend into the maxillary sinus. In more than one instance after treatment of the frontal sinus the discharge into the nose continued, and I performed a second operation upon the maxillary sinus with result of demonstrating that empyema had existed in both the chambers. I have seen several cases in consultation where, after treatment of the frontal sinus empyema, the same condition was detected in the maxillary sinus.

*Fourth.*—The occurrence of a polypoid mass on the wall of the maxillary sinus may occur in the latter stage of any disease affecting this chamber; whether these growths are at any time the exciting causes of collections of pus is uncertain. This condition is perhaps the most inveterate of any to which the maxillary sinus is subject. Fortunately, it is rare.

*Fifth.*—In the last place we have occasionally present effusion occurring in the maxillary sinus. I have not seen this condition described, and it was unknown to me prior to my custom of using transillumination as an aid to diagnosis of disease in this portion of the body.

No case has come under my notice in which I could prove that inflammation of the maxillary sinus had extended from the nasal chamber. On anatomical grounds such an extension is readily explicable. I acknowledge that the difference between the two cases here detailed and those of inflammation of high grade is only one of degree.

I remember in this connection the case of a lady of middle age, who reported to me on account of deafness due to spastic inflammation of the middle ears. There was a large posterior hypertrophy of the inferior turbinated bone on the right side and ulceration on both sides of the triangular cartilage of the septum. The disease continuing obstinate led me to make a careful inspection of the facial structure by transillumination; to my surprise, the entire right side of the face was much more opaque than the left. Pressure over the maxillary sinus above the gum on the right side yielded a dull pain which had not been complained of. The patient was of full habit and lithæmic. The opacity gradually disappeared by attention to general health and the use of alteratives.

In a second case, also of nasal disease (chronic nasal catarrh), in a lady in middle life, suspecting disease of the sinus, I made a careful examination by transillumination without result; some time afterwards, not being quite satisfied of the validity of the previous test, I repeated it, and found that the light did not pass through on the right side. In both these cases I concluded there was a fluid occupying the sinus preventing transillumination of light, which did not distend the chamber and which did not escape into the nose.

It is probable that the instances in which this effusion occurs are more numerous than we have supposed, owing to the fact that prominent symptoms are not created by the presence of the fluid. It would have been a mistake in my judgment to have opened the sinus in either of these cases.

Occasionally diseases of obscure character are located in the region of the maxillary sinus which do not readily admit of classification. A case of this character may be here outlined: a gentleman reported suffering from pain in the right side of the face in which I was unable to determine the cause. After a futile study the patient was etherized and the parts thoroughly explored. The sinus was opened in front at a point posterior to the canine fossa. Everything was found to be in a perfectly normal condition. Nevertheless, the patient rapidly recovered. Opening the sinus thus cured a pain which was centred in the region of the face.

Let me narrate a second case. Localized neuralgia of the nature of tic douloureux was confined to the tissues upon the right side of the face in a married woman aged thirty. The parts were swollen, and the entire region of the cheek, including the malar bone, was unduly prominent. The pains were intermittent, attended with heat, flushing, and convulsive movements of the muscles of expression. The skin was at all times branny, shining with excess of sebaceous secretion, and of a redder color than that over the rest of the face. There was no discharge either into the nose or the throat; the molar teeth had been futilely extracted; the first bicuspid and the incisor were dead. The test of transillumination was negative. I etherized the patient and carefully explored the parts. The sinus was opened and found to be apparently normal; the cheek tissues were dissected up as far as the lower part of the orbit, and the infraorbital nerve dissected from the point of escape from the infraorbital canal, and a portion measuring one centimetre in length excised. The sinus was opened on a line with the canine fossa and found to be empty. It was packed with iodoform gauze, and, greatly to my surprise, on the following day on removing these strips they were found to be saturated with pus. It was not possible that the pus could come formed from a perfectly normal sinus in so a short time, and I inferred that there had been pus somewhere in the sinus before operation,—probably in the posterior part, which is occasionally provided with a septum-like spur, and thus in part separated from the anterior part. The recess may have contained pus. The patient made a good recovery. There has been no return of the neuralgia, but the empyema has been a little tardy in disappearing. At this date, after a lapse of four months, the patient is entirely free from neuralgia and subject only to a very slight occasional purulent discharge from the sinus into the nose.

Syphilitic disease, of course, may be located in the maxillary sinus as well as in any other part of the economy. I simply mention the fact of the possibility of occurrence, since cases are occasionally detected. They require no special consideration.

All affections of the maxillary sinus appear to be much less frequent in the well-to-do than in the poor class of patients. Judging from my own experience, about eight per cent. of the crania obtained from dissecting-rooms exhibit evidences of inflammation of these parts. Physicians in charge of large dispensaries report that one or two cases of antral disease are generally found at any time on the list of applicants; yet dentists in large practices inform us that they almost never see diseases of the maxillary sinus. Certainly in rhinological practice they may be said to be not infrequent, and the affection should be thoroughly studied in all its phases by any one preparing himself to undertake this line of medical work.

